# Effects of Immunocastration and Amino Acid Supplementation on Yearling Fallow Deer (*Dama dama*) Testes Development

**DOI:** 10.3390/ani14010115

**Published:** 2023-12-28

**Authors:** Thoniso Chitambala, Veit Ny, Francisco Ceacero, Luděk Bartoň, Daniel Bureš, Radim Kotrba, Tersia Needham

**Affiliations:** 1 Department of Animal Science and Food Processing, Faculty of Tropical AgriSciences, Czech University of Life Sciences Prague, 16500 Prague, Czech Republic; chitambala@ftz.czu.cz (T.C.); ny_veit@yahoo.com (V.N.); ceacero@ftz.czu.cz (F.C.); kotrba@ftz.czu.cz (R.K.); 2 Department of Cattle Breeding, Institute of Animal Science, 10400 Prague, Czech Republic; barton.ludek@vuzv.cz (L.B.); bures.daniel@vuzv.cz (D.B.); 3 Department of Food Science, Faculty of Agrobiology, Food and Natural Sciences, Czech University of Life Sciences Prague, 16500 Prague, Czech Republic; 4 Department of Ethology, Institute of Animal Science, 10400 Prague, Czech Republic

**Keywords:** castration, cervid, nutrition, sperm, venison, welfare

## Abstract

**Simple Summary:**

Deer farming for venison production has incorporated castration as a means to ease the management of male spikers (handling and injuries) and prevent breeding of slaughter animals. The present study assesses immunocastration as an animal-welfare-friendly alternative to physical castration methods, together with the use of supplementary amino acids to improve the nutrition of yearling fallow deer bucks. Indicators of testes development were assessed, as well as their correlations with secondary sexual traits and dimorphic body development (including antlers), which may be affected by castration and increased nutrition. Immunocastration disrupted testes development on a microscopic level (atrophy of seminiferous tubules, decreased sperm viability, and decreased percentages of normal sperm morphology), but testes size remains a questionable indicator of vaccination success on the macroscopic level. When the effect of nutrition is considered, amino acid supplementation shows potential for enhancing the development of yearling fallow deer bucks selected for breeding, as intact deer fed amino acids had the greatest extent of testes development and sperm viability. Thus, both aspects warrant further investigation as promising tools for application in deer farming.

**Abstract:**

Forty-four fallow deer bucks (10 months old; 22.9 ± 2.4 kg) were utilized to investigate the effects of immunocastration and amino acid supplementation on testes development. Immunocastrated bucks were administered Improvac^®^ at weeks 1, 8, and 20 of this study (control group: intact males). Starting at week 8, half of each sex received rumen-protected lysine and methionine (3:1) supplementation. At slaughter (week 37/39), body size, internal fat deposits, antler size parameters, testes weight, testes surface color, cauda epididymal sperm viability and morphology, and seminiferous tubule circumference and epithelium thickness were determined. Animals with larger body sizes, greater forequarter development, and antler growth also had greater testes development. Whilst the result of immunocastration on testes size is unexpected, testes tissue showed impaired development (atrophied seminiferous tubules), decreased sperm viability, and normal morphology. Testes tissue from immunocastrated deer was less red, possibly indicating reduced blood supply. Conversely, amino acid supplementation increased testes’ redness and sperm viability, and intact males fed amino acids showed the greatest seminiferous tubule development. Thus, immunocastration may be a welfare-friendly alternative for venison production. Whilst the results support findings from the literature that testes size is not a reliable indicator of immunocastration success, this warrants further investigation in deer over different physiological development stages.

## 1. Introduction

Venison has been marketed as a high-quality meat product that is low in cholesterol and high in polyunsaturated fatty acids, proteins, and minerals [1]. This makes it appealing to consumers who demand a healthier alternative to commercial meat products and who are now more conscious of what they eat from a perceived health and environmental impact perspective. Farmed deer is currently the primary source of venison, and it is generally obtained ethically; however, there are still management issues, particularly with male fallow deer [2].

Overall, fallow deer are seen as easily manageable animals, as they adapt to different environments with low feeding and handling requirements [3,4]. The biggest challenge during their production is documented as they approach rut (late spring, early winter), which is marked by a drastic increase in testosterone production to support mating activities and fighting to establish hierarchies, thus endangering each other as well as handlers [2]. Additionally, their antlers (or spikes during early developmental stages) pose an additional challenge. Testosterone plays an important role in antler development in fallow deer, as it initiates antler growth from the pedicle (at approximately eight months of age) and finalization of antler growth (i.e., mineralization/hardening) [5]. The growth process itself is said to last 12 to 17 weeks, during which testosterone concentrations are lower than at the point of initiation and finalization of antler growth [5]. Puberty in bucks is reached between 14 and 16 months [6,7], at which point fertile and motile spermatozoa are produced [8,9]. However, in wild populations, they would initiate mating activities at approximately four years old, when they are mature enough to challenge other males during the rutting period [6]. As a photoperiodic species [10], fallow deer undergo annual cyclic changes in testes development. Luteinizing hormone (LH) release from the pituitary gland is regulated by changes in day length, with lower LH concentrations occurring during the non-breeding season and increasing in concentration as the breeding season approaches in the autumn [11]. LH promotes testes growth, activity, and testosterone production, which in turn increases testes size, spermatogenic activity, and the number of viable spermatozoa. As the rutting season ends and LH levels decrease, testosterone levels drop, and the opposite happens: testes regress and produce less spermatozoa [10].

One method for managing breeding and agonistic behavior that has been practiced since the inception of commercial fallow deer farming in New Zealand is physical castration [12], which is usually carried out at six months of age as soon as the testes descend into the scrotum [2]. This practice in the livestock industries, however, has drawn outcries from welfare enthusiasts who believe that the discomfort and side effects [13] that the animals face to meet human needs are unwarranted. Immunocastration has been suggested as an alternative to physical and surgical castration in many production systems. It is the administration of a vaccine (at least twice, depending on the production system length of the species) to an animal, which prevents the action of gonadotropin-releasing hormone (GnRH), usually by preventing it from binding to the pituitary gland (in the case of the Improvac^®^ vaccine). GnRH plays a role in the endocrine cascade, which stimulates testes development and supports functioning by initiating the release of LH and Follicle Stimulating Hormone (FSH). FSH promotes the production of sperm by aiding in the development of the seminiferous tubules, while LH aids in the production of testosterone [14,15]; however, secretion is influenced by day length and seasonality [16]. This anti-GnRH vaccine leads to a decrease in androgen hormone levels by atrophying the testes and affecting the development of the seminiferous tubules, thus interrupting the development of viable spermatozoa [15,17]. Immunocastration has helped ease the management of male livestock through the reduction of aggressive behavior [15]. However, as there is no commercial vaccine with commercial vaccination schedule guidelines for small ruminants (only for large stock, namely cattle—Bopriva^®^), there is a need to further understand the efficacy of such vaccines in other species of ruminants according to their production goals.

A comprehensive review [18] focusing on the supplementation of amino acids in cervids (and other ruminants) summarizes the roles of the first limiting amino acid, lysine (Lys), and methionine (Met) in the improvement of growth rates and body and reproductive development (sperm quality as well as fertility) of ruminants. For example, previous studies have shown that the supplementation of various deer species with different rumen-protected amino acids increases body weight gains [19,20], improves the meat yield of high-value cuts, and decreases internal fat deposition in fallow deer used for venison [21]. In breeding animals, rumen-protected amino acids have been shown to improve the sperm mass activity, motility, concentration, and membrane integrity of rams [22]. Although the utilization of rumen-protected amino acid nutrition in deer farming is not well-studied, it may have potential for improving productivity under certain situations, especially the first limiting amino acid, Lys, and Met [18]. Castration of males decreases their lean growth potential due to the absence of androgens, typically increasing fat deposition and decreasing lean muscle growth [23]. Thus, it seems necessary to adjust the nutrition of castrated animals to ensure that excess nutrients are not utilized for energy and fat storage. Similarly, immunocastration influences nutrient requirements in other species [23], including dietary balanced protein levels (according to Lys and Met) [24]. Studies have investigated the adjustment of amino acids according to the immunocastration vaccination schedules in swine to aid in supporting feed efficiency and carcass leanness [23,24]. Only one study currently exists considering the effects of immunocastration in fallow deer on blood biochemical markers linked to metabolism [25], which reports changes in protein metabolism (decreased protein anabolism after immunocastration); however, no consequential effects of this were seen on growth or slaughter performance data.

The aim of this study was to investigate the effects of immunocastration, as well as rumen-protected lysine and methionine supplementation, on testes development and functioning in yearling fallow deer bucks, as well as differential body development and secondary sexual traits, like antler development, to determine their possible benefits in deer farming.

## 2. Materials and Methods

All experimental procedures conducted during this study and the use of animals were approved by the Institutional Animal Care and Use Committee at the Czech University of Life Sciences, Prague, within its competence (Permit: 63479_2016-MZE-17214). The experiment was carried out at a private deer farm in South Bohemia, Czech Republic (49.17° N, 14.90° E; 485 m.a.s.l.).

### 2.1. Animals, Feeding, and Experimental Design

At the beginning of the experiment, forty-four male fallow deer (ten months old) were divided into two 2-hectare paddocks (balanced for body weight per treatment, average: 22.9 ± 2.4 kg). The body condition score was evaluated at the start of this study on a scale of 1 to 5 [26], and the animals ranged from 1.5 to 2.5 (average ± standard deviation: 1.9 ± 0.41). Within each paddock, the animals were then further allocated into two castration treatment groups: half were immunocastrated animals (IC; n = 22 per paddock) and half remained non-castrated, intact males (E; n = 22 per paddock). During the second month of this study, supplementation with rumen-protected amino acids was included in the diet of the animals within one paddock. Thus, four treatment combinations were utilized in this study under a factorial design with 11 animals in each treatment, as follows: non-castrated intact males without amino acid supplementation (E-Control), non-castrated intact males with amino acid supplementation (E-AA), immunocastrated males without amino acid supplementation (IC-Control), and immunocastrated males with amino acid supplementation (IC-AA).

All animals were fed a mixed grain diet of oats and wheat (90 oats:10 wheat) at 250 g per animal per day throughout the experiment. The AA supplementation was rumen-protected Lys at 6.3 g of the supplement per animal per day, which is equivalent to 4.3 g of L-Lysine HCL or 4.1 g of rumen by pass (95% rumen by pass) (LysiGem, Kemin Industry, Inc., Des Moines, IA, USA), and Met at 2.1 g per animal per day equal to 1.6 g of DL-Methionine or 1.4 g of ruminal bypass (90% rumen by pass) (Kessent, Kemin Industry, Inc., USA), following a 3:1 ratio of Lys to Met [27]. To best minimize competition and monopolization of the resource by dominant individuals [28], the mixture of grains and AA was fed in a 4.5 m long feeder. The person feeding the animals observed each feeding event to ensure all feed was consumed and to confirm that no major issues with competition existed. To avoid a pasture effect, the groups were rotated twice from one paddock to another during the experimental period. The most abundant plant species in the paddocks were *Lolium perenne*, *Cynosurus cristatus*, common grass species, including *Agrostis* sp., *Festuca* sp., *Poa* sp., and *Trifolium repens*, and weed species, including *Urtica dioica* and *Cirsium arvense*. Pasture samples from both paddocks were collected three times during the study period to analyze the nutrient composition. The nutrient composition of the grain mixture and pasture were analyzed [29], and the results are shown in Table 1.

**Figure 1:**
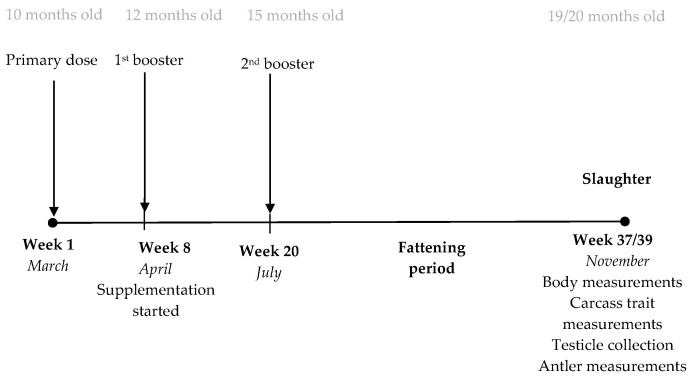
The trial timeline showing the immunocastration vaccination schedule, the feed supplementation period, and data collection at slaughter relative to the age of the deer (in grey).

The deer allocated to the immunocastrated treatment were vaccinated using three 2 mL doses of Improvac^®^ (Zoetis Animal Health, Parsippany, NJ, USA) injected subcutaneously into the area above the shoulders with a Sterimatic safety needle guard system (Sterimatic Worldwide Ltd., Gloucestershire, UK). The priming dose was injected at the beginning of the experiment in March (week 1, 10 months of age). The latter two doses acted as boosters, with the first booster applied 7 weeks after the priming dose in April (week 8, 12 months of age) and the second booster applied 3 months after the first booster dose in July (week 20, 15 months of age) (Figure 1). A second booster was utilized to ensure the duration of the immunocastration effect until slaughter based on commercial vaccination recommendations for cattle, as no recommendations are available for deer species. A vaccination interval using Bopriva^®^ (same active ingredient and concentration as Improvac^®^) of 8 weeks between the first and second vaccinations has a minimum duration effect (testosterone suppression) of 16 weeks after the second vaccination, according to the manufacturer’s guidelines.

### 2.2. Slaughtering and Carcass Measurements

After seven months of AA supplementation, all deer were slaughtered using captive bolt stunning in a restraint system, followed by exsanguination by severing the jugular veins and carotid arteries. Before slaughter, the body condition of each buck was scored (average BCS: 2.9 ± 0.30), and the live weight was also recorded (average slaughter weight: 39.55 ± 0.89 kg). The animals were slaughtered over two days, where half of the animals from each treatment group were randomly selected and slaughtered per day, according to the capacity of the abattoir facility and the management schedule of the commercial deer farm. This approach also allows for the finishing of slaughter animals before winter, when the animals would start using excess nutrition to increase their fat stores and subsequently prepare for their first rut in the season thereafter [28]. Linear measurements were taken on the carcasses, including height at the withers, body length, heel length, neck circumference, chest circumference, and leg circumference, using a flexible tape measure [30].

After the measurements, carcasses were transported for processing at the abattoir, which was located 20 km from the farm, where carcasses were eviscerated and weighed (hot carcass weight) before chilling at 4 °C. The dressing percentage was calculated as: (hot carcass weight/slaughter weight) × 100. The internal fat deposits, such as kidney fat, heart fat, and stomach fat, were removed and weighed [31]. Antler length was measured before cutting at the burr level using an electric saw and then weighed [32].

Immediately after slaughter, both testes were removed from the scrotum, and each was trimmed of excess tissues and epididymis and weighed together (trimmed testes weight) using a precision scale with accuracy ± 0.01 g (Kern, Verkon, Frankfurt am Main, Germany). Thereafter, 1 g of cauda epididymis was removed, placed in a microtube with 3 mL of physiological saline, and macerated. Then, 10 μL was pipetted onto a clean, labelled microscopic slide, followed by 20 μL of nigrosine–eosin staining media, to produce a smear slide. After drying, the percentage of dead versus alive sperm was determined by evaluating 150 sperm per sample (at 100× magnification) (Nikon Eclipse E200, Nikon, Tokyo, Japan). Additionally, the smear slides were also used to determine the percentage of normal versus abnormal sperm by evaluating 100 sperm per sample (at 100× magnification) (Nikon Eclipse E200, Nikon Japan). For sperm morphology, the head, neck, and tail parameters were assessed for abnormal morphology (100 sperm per samples). Abnormalities in the head assessed consisted of tapered, pyriform, amorphous, small, and detached heads. Neck irregularities involved bent structure and cytoplasm (proximal, central, and distal), while tail abnormalities comprised short, bent, and coiled formations.

Then, another 10 μL of the extracted fluid was further diluted with 300 μL of physiological saline. Finally, 10 μL of the diluted sample was transferred to each side of a Neubauer Chamber (0.100 mm depth, 0.0025 mm^2^), and the number of sperm cells on each of the four grids on both sides of the chambers was counted using an optical microscope (at 40× magnification) (Nikon Eclipse E200, Nikon, Japan). The final sperm concentration per 1 g of cauda epididymal tissue was then calculated.

The testes were then cut in half perpendicular to the longitudinal axis of the testis and the color of the cut surface was measured (six measurements/sample) using a calibrated portable spectrophotometer (CM-2500d, Minolta, Osaka, Japan; aperture size of 8 mm including the specular component and 0% UV; illuminant/observer of D65/10°; zero and white calibration). The results were expressed by the L* (lightness), a* (redness), and b* (yellowness) coordinates of the CIELab colorimetric space. Tissue samples were cut from each testis and preserved in 10% buffered formalin for further processing for histological evaluation.

**Figure 2:**
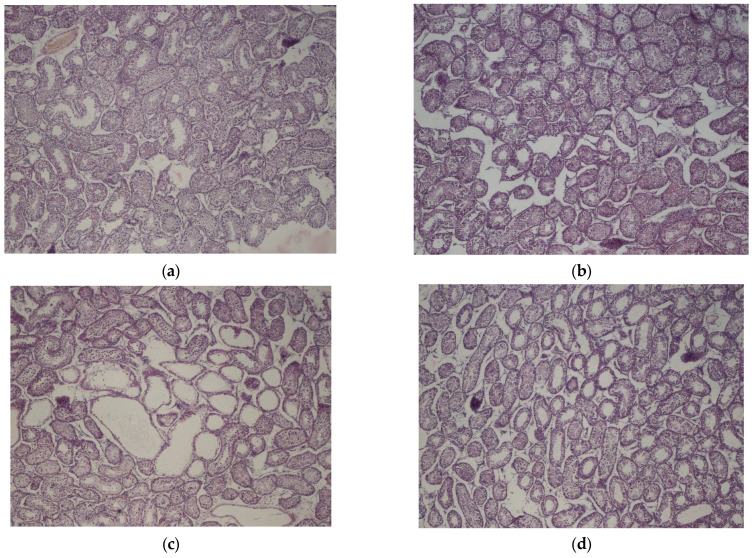
The histological images displaying cross-sections of the seminiferous tubules (40× magnification) of yearling fallow deer (approximately 19/20 months old) according to the following treatments: (**a**) entire males fed without animo acids (E-Control); (**b**) entire males fed with supplementary amino acids (E-AA); (**c**) immunocastrates fed without animo acids (IC-Control); (**d**) immunocastrates fed with supplementary amino acids (IC-AA).

The preserved testes tissue samples were washed with water and dehydrated in a graded alcohol series before being paraffin-embedded. Tissue samples were sliced into 5 μm thick sections, placed onto microscope slides, and stained with hematoxylin and eosin. The seminiferous tubule circumference and epithelium thickness of 120 seminiferous tubules per deer were measured using an optical microscope (Nikon Eclipse E200, Nikon, Japan) connected to digital ds-f1 (at 4× magnification) and the image analysis software NIS-Elements AR 3.2. (Nikon Instruments Europe B.V., Amsterdam, The Netherlands) (Figure 2).

### 2.3. Statistical Analysis

IBM SPSS Statistics 28 software was used to generate several Multivariate General Linear Models (MGLM) to assess the effects of supplementation and immunocastration treatments on the testes’ color, morphometry, and sperm quality variables. The normality of the dependent variables was tested using the Shapiro–Wilk test. Sperm concentration and viability data were not normally distributed and were thus transformed using the log transformation and square root functions, respectively. The percentage of normal sperm was utilized in the statistical analyses but for the individual sperm morphology parameters, many data points had zero values (no reported incidences of the relevant trait) with low variation in the data and thus they were not included in the statistical model. Instead, they are presented as descriptive statistics for the pooled data set. Weight and body condition were also included in the model because both variables are well-known to affect testis development, especially around puberty [14]. The Variance Inflation Factor showed no multicollinearity between body weight and two proxy variables for body condition, body condition score (BCS) and percentage of internal fat, even if the three variables were significantly correlated. Preliminary models were tested using weight and BCS or weight and internal fat; the second option consistently showed lower AIC values, and thus internal fat was selected as an indicator of body condition for building the final models. Descriptive statistics were also used to calculate estimated mean values and standard error for the variables grouped by nutrition, castration status, and the interaction between nutrition and castration status.

Relationships among the studied testes variables and between them and selected animal characteristics (withers height, body length, heel length, neck circumference, chest circumference, leg circumference, carcass weight, dressing percentage, kidney fat, heart fat, stomach fat, antler length, and antler weight) were analyzed using partial correlations. Statistical tests used the following significance levels: *p* ≤ 0.05, *p* < 0.01, and *p* < 0.001.

## 3. Results

The whole body weight at slaughter and the internal fat percentage affected the MGLM in terms of the testes’ morphometry (trimmed testes weight, seminiferous tubule circumference, and seminiferous tubule epithelium thickness) and sperm quality (sperm concentration, normal morphology %, and live sperm %), but not color (Table 2).

The interaction between castration status and nutrition was significant for the seminiferous tubule circumference as well as the percentage of normal sperm (*p* < 0.05; Table 2), with immunocastrates fed without supplementary AA (IC-Control) having the smallest seminiferous tubule circumferences (*p* = 0.05) as well as the lowest percentage of normal sperm (*p* < 0.05) present in the sample from the cauda epididymis (i.e., a higher proportion of abnormal sperm morphologies; Table 3). The analysis of sperm head defects revealed the following percentages: tapered heads at 0.9 ± 0.29%, pyriform at 0.3 ± 0.76%, amorphous at 0.7 ± 1.31%, small heads at 0.4 ± 0.72%, and detached heads at 8.0 ± 7.98%. Sperm neck defects comprised bent necks at 0.02 ± 0.15%, proximal cytoplasm at 9.8 ± 13.46%, central cytoplasm at 1.3 ± 5.36%, and distal cytoplasm at 1.4 ± 3.77%. Tail defects included short tails at 1.1 ± 2.06%, bent tails at 0.2 ± 0.91%, and coiled tails at 23.1 ± 15.2%. Immunocastration decreased seminiferous tubule epithelium thickness (*p* < 0.001; Table 2) compared to non-castrated males (Table 3). Additionally, immunocastration decreased the percentage of live sperm (*p* < 0.01; Table 2 and Table 3) and sperm with normal morphology (*p* < 0.05; Table 2 and Table 3) compared to non-castrated males. Despite this, immunocastrated deer had slightly higher trimmed testes weight (*p* < 0.05). Immunocastration increased L* values (*p* < 0.01; Table 2) compared to non-castrated males (i.e., immunocastrates had lighter testes color) (Table 3).

Nutrition affected the a* values of the cut surface testes color (*p* < 0.05; Table 2), with animals fed supplementary amino acids having higher a* values (i.e., the testes were redder; Table 3). Additionally, amino acid supplementation increased seminiferous tubule epithelium thickness (*p* < 0.01; Table 2 and Table 3). On the contrary, control animals (i.e., not fed supplementary amino acids) had higher trimmed testes weight (*p* < 0.05; Table 2 and Table 3). Furthermore, non-castrated males without AA supplementation had the highest percentage of sperm with normal morphology (Table 2; *p* < 0.05).

Seminiferous tubule circumference and thickness showed weak positive correlations with a* values (*p* < 0.05; Table 4) and weak to moderate correlations with L* values (*p* < 0.01; Table 4); i.e., testes with larger seminiferous tubule circumferences and thicker epithelium had cut surface colors that were less bright (darker) and redder. Seminiferous tubule parameters showed a strong positive correlation with each other (*p* < 0.001; Table 4), indicating that the larger the tubule circumference, the thicker the tubule epithelium. The percentage of normal sperm was negatively correlated (*p* < 0.05; Table 4) with b* values (i.e., animals with higher percentages of normal sperm had less yellow and more blue testes surface color). The trimmed testes weight showed a strong positive correlation with sperm concentration (*p* < 0.001; Table 4) and a weak positive correlation with seminiferous tubule circumference, whereas sperm concentration showed a weak positive correlation with seminiferous tubule circumference, i.e., larger, well-developed testes had larger seminiferous tubule circumferences and a higher sperm concentration.

The trimmed testes weight showed a moderate positive correlation with carcass weight, chest circumference, heel length (*p* < 0.001; Table 5), and body length (*p* < 0.01; Table 5). It also showed a weak positive correlation (*p* < 0.05; Table 5) with withers height, neck circumference, dressing percentage, and antler weight.

## 4. Discussion

This study assessed the effects of immunocastration and amino acid supplementation on the testes development of fallow deer bucks. The fallow deer’s reproductive cycle is based on seasonal changes that also influence the release of androgen hormones. Puberty in bucks is reached between 14 and 16 months, a stage at which motile, fertile spermatozoa have been observed [9], although the bucks are not used for breeding until they reach about four years of age. It is important to note that the bucks used in this study were yearlings, and many of the findings can be attributed to their age and the stage of the reproductive cycle.

Unlike some livestock breeds, testes size in fallow deer is highly related to season, and testes size increases to support spermatogenesis as they approach the breeding season (or rut) and decrease when out of season [33]. In the present study, the fallow deer were slaughtered during the period that would have been their first rut (November, early winter). Thus, comparison of information from other livestock species is complicated. In the present study, immunocastration affected seminiferous tubule development of the immunocastrated bucks, indicating tissue atrophy and thus reduced testes activity. These effects were seen in the size reduction of the circumference and epithelium of the seminiferous tubules, where spermatogenesis occurs in mammals. However, testes sizes of the immunocastrated deer within the present study were not smaller than those of the non-castrated males, as one would expect. The reasons for this are unclear and may be the result of a number of factors (e.g., changes in other physiological components), as failure of the vaccination schedule would not have resulted in significant changes in the seminiferous tubule development or sperm parameters. Future studies should try to incorporate scrotal circumference over the development of the fallow deer to better describe changes in individuals’ testes sizes, but this requires addressing some practical difficulties of measurement in a restraint crush system.

Color parameters of the deer testes surface in the present study indicated that testes with larger seminiferous tubule circumferences and thicker epithelium also had redder and darker a* values. This was the opposite for the immunocastrated deer, which had higher L* values of their testes surface color, likely indicating decreased testes activity. Additionally, those animals with higher percentages of sperm with normal morphology had less yellow (and bluer) testes color. It is possible that the link between color and reduced testes activity may be due to reduced blood supply to the testes. Similar results were found in pigs, where testes tissue atrophy was also associated with lighter, less red testes color [24,34]. However, whilst this trend is observed in other studies, the association between the color and functionality of testes needs further investigation, as information on testis surface color in most livestock species is currently limited, and it is thus difficult to draw clear conclusions in this regard.

Immunocastration also decreased the percentage of live and normal morphology sperm. It is not unexpected that sperm may still be found in the cauda epididymides of immunocastrated animals, and similar results have been found in other studies on, e.g., sheep [35,36]. Immunocastration vaccination schedules for other species typically focus on suppressing testosterone to a level low enough to prevent agonistic behaviors, and, in the case of swine, to suppress androgen production enough to allow for boar taint clearance, and thus does not rely on the complete elimination of testosterone production. Ideally, viable sperm percentages in livestock should be approximately 70% [14]. Thus, in the present study, the percentage was still quite low even in the control group, which was possibly linked to their physiological age [10]. When considering the correlation analyses, fallow deer with heavier testes had more developed seminiferous tubules (i.e., larger circumferences), increased sperm concentrations, and higher sperm concentrations, as expected. This is also observed when weight and body condition are considered within the model, so these findings are independent of these variables in this study.

Fallow deer are sexually dimorphic animals [31]. Testosterone plays an important role in antler development in male fallow deer by initiating pedicle development and antler mineralization, whilst IGF-1 is responsible for the growth process itself [37,38]. This is also true for other cervid species, including Iberian red deer [39] and white-tailed deer [17]. A greater degree of testes development in the current study was positively associated with larger body sizes, greater forequarter development, and antler development. Detailed antler quality data published from the same animals included within the present study showed that immunocastrated fallow deer had lighter (in weight) antlers, a lower amount of cortical bone development, and less mineralization (decreased density and lower calcium and phosphorous concentrations) compared to non-castrated males [32]. These results support the role of testosterone in fallow deer antler development. However, reliable monitoring of changes in serum hormone concentrations require frequent and standardized blood sampling, which is challenging in species that are difficult to handle frequently, such as fallow deer. Whilst the effects of immunocastration on serum testosterone levels have been established over a range of species, further in-depth studies should utilize samples that better represent longer-term hormone levels, such as fecal hormone metabolites (using validated assays for fallow deer [40]). Amino acid supplementation is a novel topic in deer nutrition, particularly for reproduction. Lys and Met are the most-studied AAs and also the most-limiting AAs for high-producing ruminants, necessary for the rapid growth of young ruminants [18]. Improving dietary protein and AA profiles in deer nutrition is important for growth performance and production, especially during antler growth [41,42]. A positive effect of AA supplementation was seen for seminiferous tubule circumference and epithelium thickness development and live sperm (%) in the present study. Lys has an essential role in the Lys acetylation in spermatogenesis [43], and Met is also important for the methylation function in early spermatogenesis [44]. AA supplementation has demonstrated notable improvements in various semen traits, including motility, velocity, morphologically normal sperm, and acrosome integrity in other species [45]. For instance, Lys enhances sperm quality and fertility in swine [46]. Similarly, Met has exhibited the ability to augment libido and sperm motility in rabbits [47], while sheep rams fed rumen-protected Met supplementation had increased percentages of live sperm, testes size, and serum testosterone levels [48]. Furthermore, a combination of rumen-protected Met and Lys has displayed positive effects on semen quality and testicular functioning in rams [45], showing increased sperm concentration, reduced incidence of deformed sperm, elevated testosterone levels, wider seminiferous tubule diameters, and increased Leydig cells and Sertoli cells. Additionally, in the present study, AA supplementation influenced the redness of the testes color surface, which could further indicate that AA supplementation might improve spermatogenesis activity. The indirect effect of AAs can also be evidenced through the improvement of body growth and antler growth, as deer need to reach a threshold body weight to start their antler growth (secondary sexual traits) and the onset of puberty [37,49]. As indicated in the correlations, the reproductive parameters, including seminiferous tubule circumference, thickness, and percentage of live sperm, were positively related to most linear body measurements (indicating dimorphic growth) and antler growth. Thus, in contrast to immunocastration, supplementation of rumen-protected Lys and Met could prove beneficial for yearling fallow deer who are selected for breeding purposes and not for venison production to support development and reproductive functioning later in their productive life.

## 5. Conclusions

Immunocastration impaired the development of the testes of yearling fallow deer by atrophying seminiferous tubules, affecting sperm morphology (increased abnormalities), and decreasing sperm viability. Additionally, testes color parameters were affected by immunocastration and nutrition, further motivating the investigation into the association between reproductive functionality traits and testes color as an indication of possibly differing degrees of testes activity. The effect of immunocastration on testes size supports previous reports that this is not always a good indicator of vaccination success, but this requires further investigation in fallow deer. On the other hand, rumen-protected amino acid supplementation (Lys and Met) in young male fallow deer rather improved reproductive development by increasing sperm viability, with non-castrated males fed amino acids having the greatest degree of seminiferous tubule development and percentages of normal sperm morphologies. Lastly, animals with larger testes also had larger body sizes, greater forequarter development, and antler growth, indicating some relationships between reproductive development and secondary sexual traits/body development traits. Thus, both tools (immunocastration and rumen-protected amino acid supplementation) show potential for utilization in commercial deer farming systems for venison production where suboptimal breeding males are culled for meat purposes, and immunocastration can be applied to control the various welfare issues already highlighted. In the case of selectively breeding male replacements, rumen-protected amino acid supplementation may support their further development. Immunocastration showed suppression in testes functioning and thus could have implications for the behavior of yearling fallow deer, which should be further investigated. The implementation of immunocastration in replacement of physical castration may thus help alleviate the pressure that farmers of deer face in relation to animal management from a welfare perspective.

## Figures and Tables

**Table 1:** Chemical composition of the pasture and concentrate supplemented to the deer bucks.

Composition (g/kg DM)	Concentrate Mixture	Pasture
Crude protein	18.56	12.28
Crude fat	3.58	1.04
Crude fiber	14.19	33.14
Ash	7.37	4.81
Nitrogen-free compounds	56.31	48.73
Acid detergent fiber (ADF)	13.24	35.61
Neutral detergent fiber (NDF)	35.55	69.72

**Table 2:** Multivariate GLMs showing the effects of the supplementation of amino acids, immunocastration, and their interaction on testes color, morphometry, and sperm quality in fallow deer bucks.

	n	R^2^	Nutrition	Castration	Nutrition x Castration	Body Weight	Internal Fat (%)
COLOR							
*Wilk’s λ*			0.869	0.669 **	0.779 *	0.911	0.983
L*	40	0.377	ns	**	ns	ns	ns
a*	40	0.182	*	ns	ns	ns	ns
b*	40	0.040	ns	ns	ns	ns	ns
MORPHOMETRY							
*Wilk’s λ*			0.548 ***	0.360 ***	0.845	0.446 ***	0.713 *
Testes weight (g)	39	0.601	*	*	ns	***	ns
ST circumference (µm)	39	0.507	**	**	*	**	*
ST thickness (µm)	39	0.597	**	*	ns	**	***
SPERM QUALITY							
*Wilk’s λ*			0.800	0.675 **	0.741 *	0.475 ***	0.819
Sperm concentration (cells/g)	37	0.561	ns	ns	ns	***	ns
Live sperm (%)	37	0.453	*	**	ns	ns	*
Normal-morphology sperm (%)	37	0.274	ns	*	*	ns	ns

Significance at *p* < 0.05, *p* < 0.01, and *p* < 0.001 levels is indicated by *, **, and ***, respectively. ns: non-significant; ST: seminiferous tubule.

**Table 3:** The estimated mean values and standard error (SE) of the testes color, morphometry, and sperm quality parameters from immunocastrated fallow deer bucks (n = 44), with and without (control) amino acid supplementation.

	Nutrition		Castration Status		Nutrition x Castration Status			
	AA	Control	E	IC	E-AA	E-Control	IC-AA	IC-Control
L*	62.6 ± 0.34	63.5 ± 0.34	62.4 ± 0.34	64.0 ± 0.34	62.3 ± 0.48	62.5 ± 0.49	63.0 ± 0.48	64.6 ± 0.48
a*	3.9 ± 0.14	3.5 ± 0.14	3.8 ± 0.14	3.7 ± 0.14	4.00 ± 0.20	3.6 ± 0.20	3.9 ± 0.20	3.5 ± 0.20
b*	12.7 ± 0.16	12.7 ± 0.16	12.6 ± 0.16	12.7 ± 0.16	12.6 ± 0.22	12.7 ± 0.22	12.8 ± 0.22	12.7 ± 0.22
ST circumference (µm)	490.7 ± 5.01	468.1 ± 5.14	489.7 ± 4.93	469.0 ± 5.07	493.8 ^a^ ± 7.04	485.7 ^a^ ± 7.14	487.6 ^a^ ± 7.00	450.5 ^b^ ± 7.38
ST epithelium thickness (µm)	39.4 ± 0.67	36.3 ± 0.69	40.3 ± 0.66	35.5 ± 0.68	41.5 ± 0.94	39.1 ± 0.96	37.4 ± 0.94	33.6 ± 0.99
Testes weight (g)	31.9 ± 1.07	35.1 ± 1.10	31.6 ± 1.05	35.3 ± 1.08	30.4 ± 1.50	32.9 ± 1.52	33.4 ± 1.49	37.2 ± 1.57
Sperm concentration (cells/g) ^#^	3.9 ± 0.44 × 10^8^	5.7 ± 0.72 × 10^8^	4.9 ± 0.66 × 10^8^	4.7 ± 0.58 × 10^8^	4.2 ± 0.70 × 10^8^	5.7 ± 1.13 × 10^8^	3.7 ± 0.56 × 10^8^	5.8 ± 0.96 × 10^8^
Live sperm (%) ^#^	21.7 ± 2.80	9.5 ± 1.12	22.9 ± 2.82	9.0 ± 0.84	32.9 ± 2.03	11.7 ± 1.68	10.5 ± 1.07	7.3 ± 1.14
Normal-morphology sperm (%)	51.4 ± 0.86	47.6 ± 3.41	55.4 ± 1.53	43.7 ± 2.33	50.2 ^b^ ± 1.34	61.2 ^a^ ± 0.99	52.5 ^b^ ± 1.03	33.9 ^c^ + 1.68

^abc^: indicates significant differences in the interaction terms between nutrition and castration status (*p*-values reported in Table 2). ^#^ indicates that variables were back-transformed. AA: supplemented with amino acids; Control: not supplemented with amino acids; E: intact; IC: immunocastrated; E-AA: intact and supplemented with amino acids; E-Control: intact and not supplemented with amino acids; IC-AA: immunocastrated and supplemented with amino acids; IC-Control: immunocastrated and not supplemented with amino acids.

**Table 4:** Pearson’s correlation coefficients (r) between the characteristics of testes and sperm quality parameters indicating reproductive functioning in fallow deer bucks (n = 44).

	L*	a*	b*	ST Circumference (µm)	ST Epithelium Thickness (µm)	Testes Weight (g)	Sperm Concentration (Sperm/g)
L*		−0.598 ***	0.374 *				
b*		ns					
ST circumference (µm)	−0.424 **	0.312 *	ns				
ST epithelium thickness (µm)	−0.398 **	0.337 *	ns	0.814 ***			
Testes weight (g)	ns	ns	ns	0.331 *	ns		
Sperm concentration (cells/g)	ns	ns	ns	0.322 *	ns	0.637 ***	
Live sperm (%)	ns	ns	ns	ns	ns	ns	ns
Normal-morphology sperm (%)	ns	ns	−0.315 *	ns	ns	ns	ns

Significance at *p* < 0.05, *p* < 0.01, and *p* < 0.001 levels is indicated by *, **, and ***, respectively. ns: non-significant; ST: seminiferous tubule.

**Table 5:** Pearson’s correlation coefficients (r) between the characteristics of reproduction parameters and linear body measurement, carcass traits, and antlers in fallow deer bucks (n = 44).

	Testes Weight (g)	Sperm Concentration (Sperm/g)	ST Circumference (µm)	ST Epithelium Thickness (µm)	L*	a*
Withers height (cm)	0.358 *	0.400 **	ns	ns	−0.338 *	ns
Body length (cm)	0.494 ***	0.455 **	ns	ns	ns	ns
Heel length (cm)	0.523 ***	0.405 **	ns	ns	ns	ns
Neck circumference (cm)	0.304 *	ns	0.308 *	ns	ns	ns
Chest circumference (cm)	0.656 ***	0.423 **	ns	ns	−0.461 **	ns
Leg circumference (cm)	ns	ns	ns	ns	ns	ns
Carcass weight (kg)	0.663 ***	0.698 ***	ns	ns	ns	ns
Dressing percentage (%)	0.357 *	ns	ns	ns	ns	ns
Kidney fat (%)	ns	ns	ns	ns	ns	ns
Heart fat (%)	ns	ns	−0.313 *	−0.511 ***	ns	ns
Stomach fat (%)	ns	ns	ns	ns	ns	ns
Antler length (cm)	ns	0.409 *	0.503 **	ns	−0.429 **	0.400 *
Antler weight (g)	0.390 *	0.390 *	0.577 ***	0.403 *	−0.571 ***	0.402 *

Significance at *p* < 0.05, *p* < 0.01, and *p* < 0.001 levels is indicated by *, **, and ***, respectively. ns: non-significant; ST: seminiferous tubule. Parameters, such as b*, live, and normal sperm %, did not show significant correlations with the linear body measurements or other secondary sexual traits and are therefore not shown.

## Data Availability

Data available upon request from corresponding author.
